# Advances in Metabolic Engineering of *Saccharomyces cerevisiae* for Cocoa Butter Equivalent Production

**DOI:** 10.3389/fbioe.2020.594081

**Published:** 2020-10-15

**Authors:** Mengge Wang, Yongjun Wei, Boyang Ji, Jens Nielsen

**Affiliations:** ^1^Key Laboratory of Advanced Drug Preparation Technologies, Ministry of Education, School of Pharmaceutical Sciences, Zhengzhou University, Zhengzhou, China; ^2^Department of Biology and Biological Engineering, Chalmers University of Technology, Gothenburg, Sweden; ^3^Novo Nordisk Foundation Center for Biosustainability, Technical University of Denmark, Lyngby, Denmark; ^4^BioInnovation Institute, Copenhagen, Denmark

**Keywords:** cocoa butter equivalents, *Saccharomyces cerevisiae*, metabolic engineering, synthetic biology, lipid biosynthesis

## Abstract

Cocoa butter is extracted from cocoa beans, and it is mainly used as the raw material for the production of chocolate and cosmetics. Increased demands and insufficient cocoa plants led to a shortage of cocoa butter supply, and there is therefore much interesting in finding an alternative cocoa butter supply. However, the most valuable component of cocoa butter is rarely available in other vegetable oils. *Saccharomyces cerevisiae* is an important industrial host for production of chemicals, enzyme and pharmaceuticals. Advances in synthetical biology and metabolic engineering had enabled high-level of triacylglycerols (TAG) production in yeast, which provided possible solutions for cocoa butter equivalents (CBEs) production. Diverse engineering strategies focused on the fatty acid-producing pathway had been applied in *S*. *cerevisiae*, and the key enzymes determining the TAG structure were considered as the main engineering targets. Recent development in phytomics and multi-omics technologies provided clues to identify potential targeted enzymes, which are responsible for CBE production. In this review, we have summarized recent progress in identification of the key plant enzymes for CBE production, and discussed recent and future metabolic engineering and synthetic biology strategies for increased CBE production in *S*. *cerevisiae*.

## Introduction

Cocoa butter (CB) is mainly extracted from cocoa beans of cocoa tree (*Theobroma cacao*), and is usually used as food flavor and cosmetics additive (Jahurul et al., 2013). With the economic development, global demands of chocolate and other CB-based products increase. As the main sources of CB, the cocoa tree can only grow in the tropical area with limited planting area, and the large-scale cocoa farming would occupy the rain forest space and thus threat the global food supplies (Clough et al., 2009). Moreover, the pest damage and infectious disease of cocoa tree might reduce CB yields (Drenth and Guest, 2016). Therefore, CB supply is quite limited, and an alternative CB supply is of interest.

CB is mainly composed of three different kinds of triacylglycerols (TAG), including 1,3-dipalmitoyl-2-oleoyl-glycerol (POP, C16:0–C18:1–C16:0), 1-palmitoyl-3-stearoyl-2-oleoyl-glycerol (POS, C16:0–C18:1–C18:0), and 1,3-distearoyl-2-oleoyl-glycerol (SOS, C18:0–C18:1–C18:0) (Lipp and Anklam, 1998; Vieira et al., 2015). SOS is the key CB flavor composition, and it is the most valuable composition in CB (Jahurul et al., 2013). Cocoa butter equivalents (CBEs) are lipids that have similar physicochemical properties as CB, and vegetable oils are often used as CBEs (Lipp and Anklam, 1998; Sonwai et al., 2014). Though the POP and POS contents are high in some vegetable oils, such as coconut oil and palm oil, the SOS content in vegetable oils is low (Lipp and Anklam, 1998). The properties of these vegetable oils-derived CBEs normally are different with CB, since they have lower melting temperature and the mouth feeling is different with CB (de Silva Souza and Block, 2018). Shea butter and a few other tropical butters are ideal CBEs (Zeng et al., 2020), but their supply is limited due to their limited distribution in the tropical area.

Yeasts are attractive choices for industrial-scale microbial oleochemical production (Zhou et al., 2016b; Spagnuolo et al., 2019). The accumulation of TAGs is a way for carbon storage in yeast, and the main TAGs of yeast are composed of C16 and C18 fatty acids (Klug and Daum, 2014). Many yeasts are generally recognized as safe (GRAS) species and can be used in the food and cosmetic industry, enabling yeasts as potential CBE production hosts. Several different wild-type yeast species had been used for CBE production (Wei et al., 2017b), and *Saccharomyces cerevisiae* is the most widely used microbial cell factories. However, no SOS was detected in the lipidome of *S. cerevisiae* BY4741 (Ejsing et al., 2009), and the SOS content of *S. cerevisiae* CEN.PK113-7D was low (Ejsing et al., 2009). Therefore, metabolic engineering and other synthetic biology strategies need to be applied to increase CBE production of *S. cerevisiae*, especially the SOS production. Metabolic engineering of *S. cerevisiae* for CBE or SOS production requires understanding the lipid biosynthetic pathway of *S. cerevisiae* and plants.

## Fatty Acid Biosynthesis in *S. cerevisiae*

**FIGURE 1:**
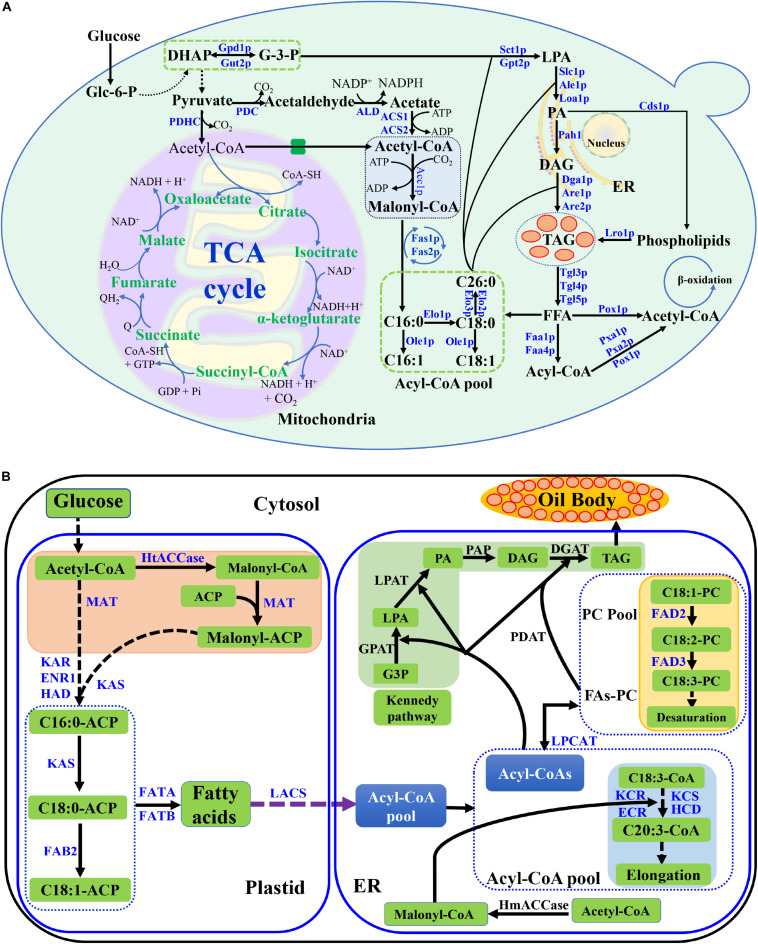
Lipid metabolic pathway in *S. cerevisiae* and lipid biosynthetic pathway in plant cell. **(A)** Lipid metabolic pathway in *S. cerevisiae*, including the biosynthesis and degradation, was described. The key enzymes were listed. Abbreviations: Glc-6-p, glucose 6 phosphate; DHAP, dihydroxyacetone phosphate; G-3-P, glyceraldehyde 3-phosphate; LPA, lysophophatidic acid; PA, phosphatidic acid; DAG, diacylglycerol; TAG, triacylglycerols; FFA, free fatty acid; TCA cycle, tricarboxylic acid cycle; PDHC, pyruvate dehydrogenase complex; PDC, pyruvate decarboxylase; ALD, acetaldehyde dehydrogenase; ACS, acetyl-CoA synthetase; ER, endoplasmic reticulum; **(B)** Lipid biosynthetic pathway in plant cell. Abbreviations: HtACCase, heteromeric acetyl-CoA carboxylase; ACP, acyl carrier protein; MAT, plastidial malonyl-CoA-ACP malonyltransferase; KAS, ketoacyl-ACP synthase; KAR, plastidial ketoacyl-ACP reductase; HAD, plastidial hydroxyacyl-ACP dehydrase; ENR1, plastidial enoyl-ACP reductase; FAB2, stearoyl-ACP desaturase; FATA, acyl-ACP thioesterase; FATB, acyl-ACP thioesterase; LACS, long-chain acyl-CoA synthetase; FAD2, ER oleate desaturase; FAD3, ER linoleate desaturase; KCS, β-Ketoacyl-CoA synthase; KCR, ketoacyl-CoA reductase; ECR, enoyl-CoA reductase; HmACCase, homomeric acetyl-CoA carboxylase; LPCAT, lysophosphatidylcholine acyltransferase; PDAT, phospholipid:diacylglycerol acyltransferase; GPAT, glycerol-3-phosphate acyltransferase; LPAT, lysophosphatidic acid acyltransferase; PAP, phosphatidic acid phosphatase; DGAT, acyl-CoA:diacylglycerol acyltransferase; G3P, glycerol-3-phosphate. Dashed arrows indicate multiple steps, and solid lines indicate a single step in the metabolic pathway.

In *S. cerevisiae*, glucose is converted into Glycerol-3-phosphate (G-3-P) through glycolysis, which is the precursor of TAG backbone. Part of G-3-P is further converted into pyruvate, which is used to synthesize acetyl-CoA by pyruvate dehydrogenase complex (PDHC) in mitochondria (Krivoruchko et al., 2015). Most acetyl-CoA generated in mitochondria is consumed in the tricarboxylic acid (TCA) cycle. In the cytosol, the pyruvate dehydrogenase (PDH) bypass converts pyruvate to acetyl-CoA via three steps catalyzed by pyruvate decarboxylase (PDC), acetaldehyde dehydrogenase and two acetyl-CoA synthetase (ACS1 and ACS2) (Figure 1A; Zhang et al., 2018).

In an initial step, acetyl-CoA is carboxylated by the addition of CO_2_ to malonyl-CoA with Acetyl-CoA carboxylase (Acc1p). Malonyl-CoA is used as building blocks, and acetyl-CoA is used as the precursor in the fatty acid biosynthesis. The fatty acid biosynthesis is catalyzed by fatty acid synthase, a multienzyme complex consisting of Fas1p and Fas2p. The fatty acid types of *S. cerevisiae* are determined by the genetic background, carbon source and other effects, which usually contain C16 and C18 fatty acids with one or none double bond (Wei et al., 2017b). The *ACC*1, *FAS1*, and *FAS2* in wild-type yeast strains were replaced with same strong constitutive promoter in order to produce fatty acid consistently (Tang et al., 2015). The fatty acyl-CoA length is decided by the elongases of Elo1p, Elo2p, and Elo3p (Tehlivets et al., 2007). Usually, Elo1p controls medium length-chain fatty acid synthesis (C12 to C16), while Elo2p and Elo3p are responsible for long-chain fatty acid synthesis (up to C26) (Rossler et al., 2003; Wenning et al., 2017). Approximately 70–80% of the total fatty acids in yeasts are monounsaturated fatty acid in a wide range of cultivation conditions, which are synthesized from saturated fatty acyl-CoA precursors by the △9-fatty acid desaturase of Ole1p (Martin et al., 2007).

## Tag Biosynthesis in *S. cerevisiae*

Phosphatidic acid (PA) is a vital component in the acylglycerol lipid metabolism. PA is synthesized by two different pathways, the G-3-P pathway and the dihydroxyacetone phosphate (DHAP) pathways (Figure 1A). The conversion between G-3-P and DHAP can reversely be catalyzed by glycerol-3-phosphate dehydrogenase (Gpd1p and Gut2p). The acyl-CoA is transferred to the *sn*-1 position of glycerol-3-phosphate (G-3-P) to form 1-acyl-G-3-P (LPA) which is mainly catalyzed with the enzyme of acyl-CoA:glycerol-*sn*-3-phosphate acyl-transferase (GPAT, Sct1p, and Gpt2p) or to DHAP which is catalyzed with acyl-CoA:DHAP acyltransferase (DHAPAT). G-3-P pathway is the main pathway for lysophosphatidic Acid (LPA) formation in *S. cerevisiae* (Klug and Daum, 2014; Fakas, 2017). Subsequently, an acyl chain was added to the *sn*-2 position by lysophophatidate acyl-transferase (LPAT, Slc1p, Ale1p, or Loa1p) to yield PA. PA can either be dephosphorylated to diacylglycerol (DAG) by phosphatidic acid phosphatase (Pah1p), or converts to phospholipids by Phosphatidate cytidylyltransferase (Cds1p). Phospholipids can further convert to TAGs via the acyltransferase Lro1p. DAG is the substrate for TAG synthesis by acyl-CoA:diacylglycerol acyl-transferase (DGAT) of Dga1p (Coleman and Lee, 2004; de Kroon et al., 2013). Besides, PA can also be used for other lipids synthesis, such as phosphatidylcholine (PC), phosphatidylserine (PS), and phosphatidylinositol (PI) (de Kroon et al., 2013). TAG can be degraded to free fatty acids (FFA) with triacylglycerol lipase (Tgl3p, Tgl4p, and Tgl5p). The FFA is further converted to acetyl-CoA via Faa1p, Faa4p, Pox1p and other enzymes in peroxisome (Figure 1A).

## Lipid Biosynthesis in Plant

The *de novo* lipid biosynthetic pathway of plant and *S. cerevisiae* is different (Figure 1; Bates et al., 2013; Fakas, 2017). In plant, the major biochemical reactions for TAG biosynthesis includes plastid fatty acid synthesis step, acyl editing step and TAG synthesis step, which mainly occur in plastid, mitochondria and endoplasmic reticulum (ER). The precursors for plant fatty acid synthesis are Acetyl-CoA and malonyl-ACP, which are converted to fatty acids-ACP through catalysis by ketoacyl-ACP synthase (KAS) and several other enzymes in plastid and mitochondria (Baud and Lepiniec, 2010; Bates et al., 2013). The fatty acid ACP are further converted to fatty acids via ACP thioesterase (FATA and FATB) (Baud and Lepiniec, 2010; Argout et al., 2011). The long-chain acyl-CoA synthetase catalyzes fatty acids to acyl-CoA in ER. The acyl-CoA are further elongated or desaturated with several enzymes. The final TAG products are formed via Kennedy pathway, and the main enzymes are GPAT, LPAT, PAP, and DGAT in ER (Figure 1B). The TAGs are stored in plant oil body.

## Engineering of SOS Production in Yeast

The C18 content are lower than C16 content in *S. cerevisiae* (Ejsing et al., 2009). SOS is composed with one glycerol backbone, two steric acids (C18:0), and one oleic acid (C18:1). In order to increase CBE production in *S. cerevisiae*, the current efforts focused on: (1) increasing fatty acids especially C18 compositions via directing metabolic flux toward lipid synthesis; (2) overexpression of acyl-CoA transferases specially for CBE synthesis.

The Acetyl-CoA carboxylase Acc1p can convert acetyl-CoA to malonyl-CoA, and the *ACC1* expression level affects fatty acid composition in *S. cerevisiae*. By overexpression of *ACC1*, *FAS1*, and *FAS2*, TAG production increased four-fold over the control strain (Runguphan and Keasling, 2014). In another study, *ACC1* variant *ACC1*^S659A S1157A^ (ACC1**) can abolish *snf1* regulation and increased fatty acid production (Shi et al., 2014). Expression of ACC1** can increase TAG production, especially increased C18 composition and decreased C16:1 composition (Bergenholm et al., 2018). The Elo1p is responsible for the elongation of C16 and C18 fatty acids, while Elo2p and Elo3p are responsible for very long chain fatty acids biosynthesis. The overexpression of *ELO1* increase C18:1 titer. However the TAG content didn’t change. Combination of engineering ACC1**, *OLE1* and *ELO1* significantly increases TAG content and SOS content 5.8-fold and 48-fold, respectively (Bergenholm et al., 2018).

Several yeast fatty acid production platforms for oleochemics have been established (Supplementary Table S1; Li et al., 2014; Runguphan and Keasling, 2014; Leber et al., 2015; Zhou et al., 2016a,b; Dai et al., 2018; Ferreira et al., 2018a). Strengthening fatty acid biosynthetic pathway and weakening the degradation pathway increased C18 compositions and decreased C16:1 composition, and the final FFA titer reached 10.4 g/L (Zhou et al., 2016b). Further relieving the side-pathway competition by harnessing yeast peroxisomes increased the production of fatty-acid-derived chemical of fatty alcohols, alkanes and olefins up to seven-fold (Zhou et al., 2016a). By simplifying the lipid metabolic network with the redirection of fatty acid metabolism and the reduction of feedback regulation, a strain with 129 mg⋅g DCW^–1^ free fatty acid production was achieved (Ferreira et al., 2018b). Moreover, by global rewiring of cellular metabolism and adaptive laboratory evolution, the Crabtree effect of *S. cerevisiae* can be abolished, which is help for the acetyl-CoA derived product accumulation (Dai et al., 2018). The *S. cerevisiae* strain was reprogrammed from alcoholic fermentation to lipogenesis, and the final FFA titer reached 33.4 g/L (Yu et al., 2018). Overexpression of *ACC1*^∗∗^, *PAH1* and DGA1, and disruption of *TGL3*, *TGL4*, *TGL5*, *ARE1*, POX1, and PXA1, lead to the final TAG accumulation of 254 mg⋅g DCW^–1^, reaching 27.4% of the maximum theoretical yield in *S. Cerevisiae*, which is the highest TAG titer reported in *S. cerevisiae* (Ferreira et al., 2018a).

GPAT, LPAT, and DGAT are the key enzymes in CBE/SOS production (Maraschin et al., 2019). The identification of plant SOS biosynthetic genes and their expression in *S. cerevisiae* can enhance SOS production (Wei et al., 2017a, 2018). There are two GPAT (Gat1p and Gat2p) and one DHAPAT in *S. cerevisiae*. The double deletions of both *GAT1* (also known as *GPT2*) and *GAT2* (also known as *SCT1*) leads to yeast lethality, showing that Gat1p and Gat2p are essential in TAG synthesis (Zheng and Zou, 2001). The acyl-specificity of Gat1p is diverse, as it can use a broad range of fatty acids as substrates; while the Gat2p displayed preference toward C16 fatty acids, suggesting that Gat1p and Gat2p can’t be used to synthesize large amount of SOS directly in *S. cerevisiae* (Zheng and Zou, 2001). Plants usually contains three different types of GPAT genes, which are located in the plastid, mitochondria or cytoplasm (Yang et al., 2012). Among the 10 GPAT genes of *Arabidopsis thailiana*, GPAT-4, -6 and -8 genes strongly preferred C16:0 and C18:1 ω-oxidized acyl-CoAs over other substrates, providing hints that these GPAT genes might be responsible for SOS production in plants (Yang et al., 2012).

LPAT (EC 2.3.1.51) is believed to have the highest substrate specificity. In *S. cerevisiae*, LPATs were identified to acylate LPA with a range of different acyl-CoAs, including C18:1, C22:1, and C24:0-CoA. Introducing LPAT genes from *S. cerevisiae* into *Arabidopsis* resulted in 8% to 48% increasement of very-long-chain fatty in TAGs, showing that yeast LPAT genes would not be suitable for CBE production (Zou et al., 1997). Diverse LPATs that acylate the sn-2 of LPA to form PA had been identified, and eight LPAT genes were found in cocoa genome, which can be classified into three different clusters based on amino acid identities (Argout et al., 2011). Expression of some LPAT genes can significantly increase TAG production in *S. cerevisiae* (Wei et al., 2017a, 2018).

As previously described, two main pathways are responsible for TAG production from DAG in *S. cerevisiae*. Phospholiipid:diacylglycerol acyltransferase (PDAT, EC 2.3.1.158) uses PLs as acyl donors, and it distributes in yeast and plants (Liu et al., 2012). DGAT catalyzes the last step of TAG biosynthesis from DAG and acyl-CoA. DGAT is an acyl-CoA dependent enzyme, which catalyzes the final and only committed step of the Kennedy pathway. DGAT is essential for TAG biosynthesis in seed. Overexpression of the yeast diacylglycerol acyltransferase (DGA1) can lead to TAG production increasement in the Δ*snf2* disruptant of *S. cerevisiae* (Kamisaka et al., 2007). Two different families of DGAT1 and DGAT2 are available in yeast, plants and animals, and DGATs from different species show diversal substrate preferences (Yen et al., 2008). For example, in *A. thaliana*, DGAT1 displays preference to C16:0, while DGAT2 displays preference to C16:1 (Aymé et al., 2014). The Overexpression of four DGAT1 genes from *Brassica napus* in *S. cerevisiae* increased TAG biosynthesis (Greer et al., 2015). Moreover, expression of *Arabidopsis* DGAT gene in yeast increased 200–600 folds of yeast DGAT activity, which can lead to 3–9 folds TAG increasement, showing DGAT might be a useful target for CBE accumulation in *S. cerevisiae* (Bouvier-Navé et al., 2000).

Global genomic and transcriptomic analyses identified thirteen potential GPATs, eight potential LPATs and two DGATs in *T. cacao* (Wei et al., 2017a, 2018). The overexpression of single or multiple cocoa genes in *S. cerevisiae* increased CBE production. The combinational expression of GPAT, LPAT, and DGAT genes showed 134-fold production of TAG over the control strain, showing these genes from cocoa have great potential for CB production (Supplementary Table S1; Wei et al., 2018). Shea butter contains high-level SOS, and their GPATs, LPATs, and DGATs had been identified through the transcriptomics analyses and the functional heterologous expression in *S. cerevisiae* (Wei et al., 2019). In the future, mining more specific and efficient enzymes for CBE production would help to increase CBE composition in *S. cerevisiae*.

## Plant Gene Mining and Their Applications for Increased CBE Production

**FIGURE 2:**
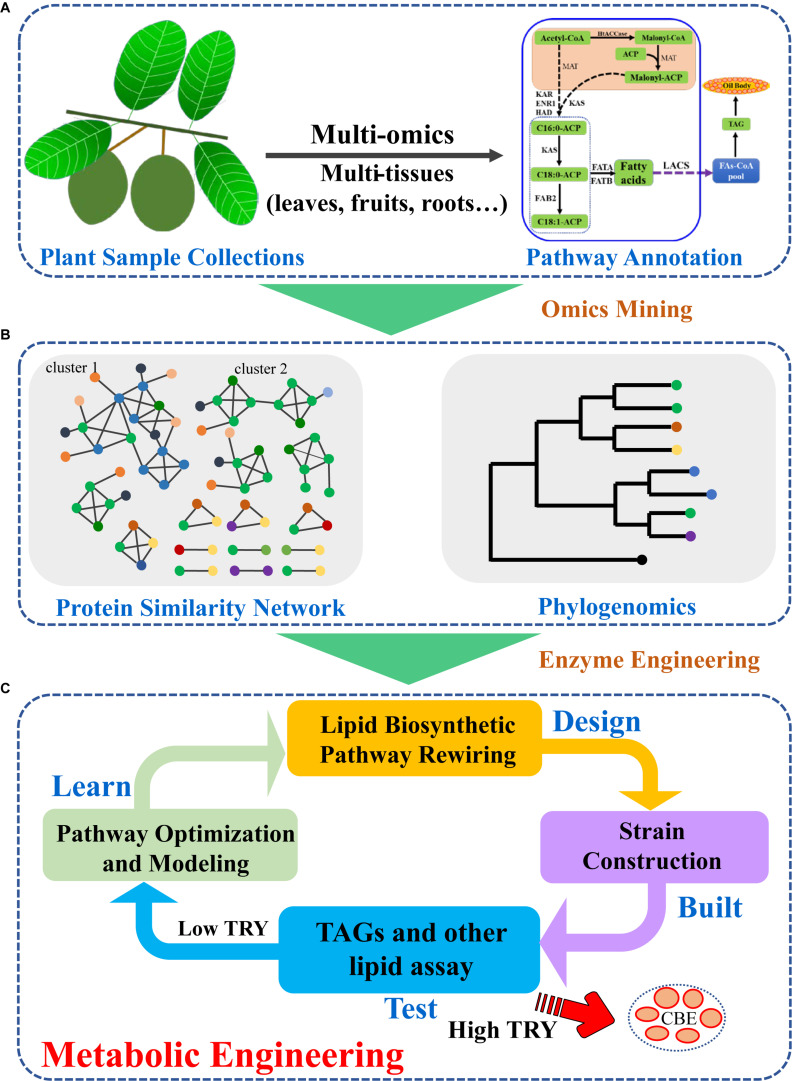
Strategies for plant TAG biosynthetic enzyme mining and metabolic engineering for increased CBE production. **(A)** Plant sample collection and multi-omics integration analyses identified potential genes in plant lipid biosynthetic pathway; **(B)** Phylogenetic and protein similarity network analyses led to efficient enzyme selection; **(C)** Classic design-build-test-learn cycle enhanced CBE production in *S. cerevisiae* by introduction of efficient plant lipid biosynthetic enzymes.

With the development of sequencing technologies, huge amounts of plant genomic or transcriptomic data had been generated (Leebens-Mack et al., 2019). The strategy to identify novel key CBE or other natural product biosynthetic genes had been extensively developed and applied (Wang et al., 2015). In general, plant samples were collected, and genome and/or transcriptome were obtained by next-generation or third generation sequencing technologies. The genome or transcriptome were assembled and annotated (Figure 2A). Plants usually harbor several enzymes for one biochemical reaction, therefore, quick identification of key enzymes for CBE production are essential. For CBE production, the potential genes involved in TAG biosynthesis can be identified based on the similarity or phylogeny (Wei et al., 2017a). Sequence similarity network, which clustered sequences based on pairwise similarity can be applied to classify sequences into subgroups. While phylogenetic analysis can give more direct insights into the relationships between newly identified genes and characterized genes. Based on phylogeny, potential CBE production genes can be identified via their neighboring characterized genes (Figure 2B). Several GPATs, LPATs, and DGATs had been identified from multi-omics data with such strategy (Wei et al., 2017a, 2018). In previous studies, cocoa GPAT, LPAT, and DGAT genes that are similar to the characterized genes or different from the known enzymes with C16 as preferred substrates were selected for experimental measurement in yeast (Wei et al., 2017a). The identified efficient/potential genes can then be used for further metabolic engineering or synthetic biology modification of *S. cerevisiae*. During the typical metabolic engineering cycle of Design-Build-Test-Learn (DBTL), lipid biosynthetic pathway can be redesigned and rewired (Nielsen and Keasling, 2016; Ko et al., 2020; Figure 2C). The designed pathway can be further built with advanced synthetic biology and systems biology tools to generate strains with high-level CBE production. When the TAG/CBE titer, rate and yield (TRY) of the engineered strains are high enough, they will be used for further large-scale fermentation; if the TRY are low, novel engineering strategies should be used for next round strain optimization until high TRY are obtained (Nielsen and Keasling, 2016; Ko et al., 2020).

## Conclusion and Perspective

Based on the high-fatty acid production *S. cerevisiae* platform strains (Ferreira et al., 2018a; Yu et al., 2018), metabolic engineering to increase specific TAG productivity by introducing efficient enzymes of GPAT, LPAT, and DGAT would benefit CBE production. With the development of DNA sequencing technology, the whole-genome sequencing and transcriptomics of numerous plants have enabled systematic analysis of TAG and lipid production pathways in different plant species, which has provided the basis for future screening of efficient GPAT/LPAT/DGAT candidates (Cheng et al., 2018). By integrating the transcriptomics and lipidomics data, it will help to identify specific enzymes engaged in the production of targeted TAGs. Furthermore, in terms of engineering yeast, the enhancement of acetyl-CoA and malonyl-CoA pool by disruption of the PDC genes or engineering key fatty acid biosynthetic genes (*ACC1*, *FAA1*, *FAA4*, *FAS*, et al.), enhancing/balancing cofactor of NADPH supply, down-regulating completing pathways, harnessing yeast sub-organelle metabolism will increase CBE production in engineered *S. cerevisiae* (Shi et al., 2014; Zhou et al., 2016a,b; Dai et al., 2018; Yan and Pfleger, 2020). The TAG lipases encoded by *TGL3* and *TGL4* genes had been confirmed to involve in the TAG degradation (Dulermo et al., 2013). The blockage of TAG entering into the degradation pathways could also be an efficient strategy. Moreover, appropriate low-cost substrates (such as xylose and other lignocellulose components) can also be used to reduce microbial CBE production cost in the future (Hou et al., 2017). The adaptive laboratory evolution might help to increase strain adaptation to recalcitrant substrates, in order to produce high-level fatty acid-derived chemicals from recalcitrant substrates or one carbon source of CO_2_/CH_4_, which might be applied in the future CBE production (Pereira et al., 2019; Cao et al., 2020; Liu et al., 2020; Zhu et al., 2020). Considering oleaginous yeasts can produce high-level lipids naturally (Wei et al., 2017b), metabolic engineering of selected oleaginous yeasts can be an alternative choice in the future (Kamineni and Shaw, 2020; Wang et al., 2020).

Biosynthesis of CBE using engineered *S. cerevisiae* is one promising way to satisfy growing CB demand. Efficient genes of GPATs, LPATs, and DGATs encoding for SOS production from oil crops can be screened using both computational and experimental approaches. The expression of these lipid biosynthetic genes in *S. cerevisiae* chasis with high-level C18:0- and C18:1-production ability would strongly increase the CBE production. Metabolic engineering and rewiring have enabled turning *S. cerevisiae* from alcoholic fermentation to lipogenesis, and further systems biology, synthetic biology, evolutionary engineering and other advanced systems metabolic engineering strategies might further increase CBE production in *S. cerevisiae*.

## Author Contributions

JN and YW conceived the study. MW, BJ, and YW drafted the manuscript. JN revised the manuscript. All authors contributed to the article and approved the submitted version.

## Conflict of Interest

The authors declare that the research was conducted in the absence of any commercial or financial relationships that could be construed as a potential conflict of interest.
