# Supplementary material for: Advances in Metabolic Engineering of Saccharomyces cerevisiae for Cocoa Butter Equivalent Production
Source: Front Bioeng Biotechnol. 2020 Oct 15;8:594081. doi: 10.3389/fbioe.2020.594081 (PMC7594527; doi:10.3389/fbioe.2020.594081)
Supplement: Supplementary file 1 [file Table_1.DOCX]

Table S1 Summary of free fatty acids and triacylglycerol production from carbon sources using *Saccharomyces cerevisiae*.

| Product | S. cerevisiae strains | Genetic engineering | Substrate | Scale | Titer (g/L) | Yield (g/g glucose) | Yield (% theoretical yield) | Citations |
| --- | --- | --- | --- | --- | --- | --- | --- | --- |
| Free fatty acids | MG005 | MATa, ura3-52, lys2-801, ade2-101, trp1-D63, his3-D200, leu2-D1 DFAA1::loxP, DADH1::loxP-KanMX4-loxP | 20 g/L glucose, minimal medium | Shake flask | 0.14 | 0.007 | 2 | Li, X., et al. 2014 |
| Free fatty acids | WRY1 ΔFAA1 ΔFAA pESC-Leu2d-‘TesA | Mat α; his3Δ1; leu2Δ0; lys2Δ0; ura3Δ0; faa1Δ; faa4Δ; acc1::PTEF1-ACC1;fas1::PTEF1-FAS1;fas2::PTEF1-FAS2; pESC-Leu2d-‘TesA | 20 g/L glucose, minimal medium | Shake flask | 0.4 | 0.02 (0.2%glucoseand1.8%galactose) | 6 | Runguphan, W. and J. D. Keasling (2014). |
| Free fatty acids | BY4741ΔFAA1ΔFAA4ΔFAT1ΔFAA2ΔPXA1ΔPOX1 pBTEF1-DGA1-TGL3 | BY4741 faa1Δ; faa4Δ; fat1Δ; faa2Δ; pxa1Δ; pox1Δ pBTEF1-DGA1-TGL3 | 20 g/L glucose, YPD | Shake flask | 2.2 | < 0.11 | 32 | Leber, C., et al. 2015 |
| Free fatty acids | YJZ47 | MATa MAL2‐8c SUC2 hfd1Δ pox1Δ faa1Δ faa4Δ his3Δ::HIS3+(TPIp‐MmACL‐FBA1t)+(TDH3p‐RtME‐CYC1t)+(tHXT7p‐’MDH3‐TDH2t)+(PGK1p‐CTP1‐ADH1t)+(TEF1p‐‘tesA‐HIS3t) ura3Δ::(TPIp‐RtFAS1‐FBA1t)+ (TEF1p‐RtFAS2‐CYC1t)+amdSym acc1::KlURA3+TEF1p+ACC1 | 20 g/L glucose, minimal medium | Shake flask | 1 | 0.05 | 14 | Zhou, Y. J., et al. 2015 |
| Free fatty acids | YJZ47 | MATa MAL2‐8c SUC2 hfd1Δ pox1Δ faa1Δ faa4Δ his3Δ::HIS3+(TPIp‐MmACL‐FBA1t)+(TDH3p‐RtME‐CYC1t)+(tHXT7p‐’MDH3‐TDH2t)+(PGK1p‐CTP1‐ADH1t)+(TEF1p‐‘tesA‐HIS3t) ura3Δ::(TPIp‐RtFAS1‐FBA1t)+ (TEF1p‐RtFAS2‐CYC1t)+amdSym acc1::KlURA3+TEF1p+ACC1 | minimal medium | Fed batch | 10.4 | 0.034 | 9 | Zhou, Y. J., et al. 2015 |
| Free fatty acids | Y&Z036 | MATa MAL2‐8c SUC2 ura3‐52 hfd1Δ pox1Δ faa1Δ faa4Δ his3Δ::HIS3+(TPIp‐MmACL‐FBA1t)+(TDH3p‐RtME‐CYC1t)+(tHXT7p‐’MDH3‐TDH2t)+(PGK1p‐CTP1‐ADH1t)+(TEF1p‐‘tesA‐HIS3t) ura3Δ::(TPIp‐RtFAS1‐FBA1t)+ (TEF1p‐RtFAS2‐CYC1t)+amdSym p416::URA3; X1-5::Cas9; acc1:: TEF1p-ACC1; pyc1::TEF1p-PYC1; X1-4:: MPC1+MPC3; gal80∆; X1-2:: AnACL; gal1∆gal7∆gal10∆:: RtCIT1+IDP2+YHM2; pgi1∆:: COX9p-PGI1+GND1+TKL1+TAL1+ZWF1; idh2∆:: GSY1p-IDH2; pTao06; | minimal medium | Fed batch | 33.4 | 0.1 | 30 | Yu, T., et al. 2018 |
| Triacylglycerols | RF11 | MATa ura3–52 can1::cas9-natNT2 HXT7p-ACC1** PGK1p-PAH1 TEF1p-DGA1 tgl3/4/5Δ are1Δ pox1Δ gut2Δ pxa1Δ | 20 g/L glucose, minimal medium | Shake flask | 1.76 | 0.088 | 27.4 | Ferreira, R., et al. 2018. |
| CBE | Y29-441 | IMX581 sct1Δ ale1Δ lro1Δ dga1Δ pBS01A-TcGPAT4-TcLPAT4-TcDGAT1 | 20 g/L glucose, minimal medium | Shake flask | 0.03 mg/g DCW | ND | ND | Wei, Y., et al. 2018 |

**References**

Li, X., D. Guo, Y. Cheng, F. Zhu, Z. Deng and T. Liu (2014). "Overproduction of fatty acids in engineered Saccharomyces cerevisiae." Biotechnology and Bioengineering **111**(9): 1841-1852.

Runguphan, W. and J. D. Keasling (2014). "Metabolic engineering of Saccharomyces cerevisiae for production of fatty acid-derived biofuels and chemicals." Metabolic Engineering **21**: 103-113.

Leber, C., B. Polson, R. Fernandez-Moya and N. A. Da Silva (2015). "Overproduction and secretion of free fatty acids through disrupted neutral lipid recycle in Saccharomyces cerevisiae." Metabolic Engineering **28**: 54-62.

Zhou, Y. J., N. A. Buijs, Z. Zhu, J. Qin, V. Siewers and J. Nielsen (2016). "Production of fatty acid-derived oleochemicals and biofuels by synthetic yeast cell factories." Nature Communications **7**

Yu, T., Y. J. Zhou, M. Huang, Q. Liu, R. Pereira, F. David and J. Nielsen (2018). "Reprogramming Yeast Metabolism from Alcoholic Fermentation to Lipogenesis." Cell **174**(6): 1549-1558 e1514.

Ferreira, R., P. G. Teixeira, M. Gossing, F. David, V. Siewers and J. Nielsen (2018). "Metabolic engineering of Saccharomyces cerevisiae for overproduction of triacylglycerols." Metabolic Engineering Communications **6**: 22-27.

Wei, Y., D. Bergenholm, M. Gossing, V. Siewers and J. Nielsen (2018). "Expression of cocoa genes in Saccharomyces cerevisiae improves cocoa butter production." Microbial Cell Factories **17**(1): 11.
